# HLA-C*⁣*^*∗*^0304 Associates With Beneficial Gut Microbiota and Later Onset of Type 1 Diabetes in Pediatric Cohorts

**DOI:** 10.1155/pedi/3013063

**Published:** 2025-10-28

**Authors:** Zhenran Xu, Xiaojing Li, Xiaoxiao Yuan, Chengjun Sun, Miaoying Zhang, Ruimin Chen, Haiyan Wei, Linqi Chen, Hongwei Du, Guimei Li, Yu Yang, Xiaojuan Chen, Lanwei Cui, Xin Fang, Jing Wu, Qiuyue Li, Feihong Luo

**Affiliations:** ^1^Department of Pediatric Endocrinology and Inherited Metabolic Diseases, Children's Hospital of Fudan University, National Children's Medical Center, Shanghai, China; ^2^Department of Endocrinology and Inherited Metabolic, Fuzhou Children's Hospital of Fujian Medical University, Fuzhou, China; ^3^Department of Endocrinology and Inherited Metabolic, Children's Hospital Affiliated to Zhengzhou University, Zhengzhou, China; ^4^Department of Endocrinology and Inherited Metabolic, Children's Hospital of Soochow University, Suzhou, China; ^5^Department of Pediatric Endocrinology and Inherited Metabolic Diseases, The First Hospital of Jilin University, Changchun, China; ^6^Department of Pediatric Endocrinology, Shandong Provincial Hospital Affiliated to Shandong First Medical University, Jinan, China; ^7^Department of Endocrinology and Inherited Metabolic, The Affiliated Children's Hospital of Nanchang University, Nanchang, China; ^8^Department of Endocrinology, Genetics and Metabolism, The Children's Hospital of Shanxi Province, Taiyuan, China; ^9^Department of Pediatrics, The First Affiliated Hospital of Harbin Medical University, Harbin, China; ^10^Department of Pediatrics, Fujian Medical University Union Hospital, Fuzhou, China

**Keywords:** gut microbiota, human leukocyte antigen, type 1 diabetes

## Abstract

**Objective:**

To investigate whether human leukocyte antigens (HLAs) influence gut microbiota composition and contributes to delayed type 1 diabetes mellitus (T1DM) onset in children.

**Methods:**

This multicenter cross-sectional study included 106 newly diagnosed pediatric T1DM patients (age <18 years) and 69 healthy controls from nine Chinese cities. Gut microbiota was profiled via whole-metagenome shotgun sequencing, and HLA alleles were genotyped by PCR sequence-based typing. Participants were stratified by HLA-risk scores. Statistical analyses included α/β-diversity metrics, linear discriminant analysis effect size analysis (LEfSe), and Spearman correlation adjusted for confounders.

**Results:**

Principal coordinates analysis (PCoA) exposed discernible disparities in gut microbiota structures within the high-HLA-risk T1DM cohort relative to both high- and low-HLA-risk control groups (*R*^2^ = 0.0562, *p*=0.003 and *R*^2^ = 0.0343, *p*=0.003). HLA-C^*∗*^0304 carriers exhibited delayed T1DM onset compared to noncarriers (adjusted *R*^2^ = 0.225, *p*=0.017). High-HLA-risk T1DM patients showed distinct microbiota divergence from controls (*R*^2^ = 0.0562, *p*=0.003), driven by reduced Lachnospiraceae and *Blautia* (butyrate producers) in noncarriers. Conversely, HLA-C^*∗*^0304-positive T1DM patients had enriched *Blautia* (*p*=0.005) and Lachnospiraceae (*p*=0.039), alongside lower opportunistic pathogens (*Citrobacter*; *p* < 0.05). High-HLA-risk patients also displayed lower fasting C-peptide levels than low-risk counterparts (0.19 ± 0.14 vs. 0.26 ± 0.19 µg/mL, *p*=0.029).

**Conclusions:**

Our study demonstrates that specific HLA class I subtypes (e.g., C^*∗*^0304) may modulate T1DM onset through selective enrichment of beneficial gut microbiota. Elucidating the mechanisms by which HLA variants regulate mucosal immunity and coordinate HLA-microbiota-immune interactions holds significant potential for developing targeted interventions against T1DM pathogenesis.

## 1. Introduction

Type 1 diabetes mellitus (T1DM) is characterized by insulin deficiency due to an autoimmune attack on the pancreatic islet β-cells [1]. The etiology of T1DM is multifactorial, involving both genetic and environmental factors [1, 2]. The human leukocyte antigen (HLA) region, particularly the class II HLA region, is the principal genetic determinant of T1DM susceptibility. Notably, the DRB1*⁣*^*∗*^0301-DQA1*⁣*^*∗*^0501-DQB1*⁣*^*∗*^0201 and DRB1*⁣*^*∗*^04-DQA1*⁣*^*∗*^0301-DQB1*⁣*^*∗*^0302 haplotypes are associated with an elevated T1DM risk in both Caucasian and Chinese populations [3, 4]. Beyond class II HLA alleles, recent findings emphasized the significant association of all class I HLA loci with T1DM, suggesting their role in disease development either independently or through linkage disequilibrium with class II alleles [5]. Furthermore, the influence of class I HLA alleles, such as HLA-A^*⁣*^*∗*^^1101 and C^*∗*^1502, on the onset age of T1DM highlights the importance of examining their role in disease pathogenesis [6]. With the advancement in understanding T1DM's genetic susceptibility, genetic risk scores encompassing both HLA and non-HLA single nucleotide polymorphisms have been developed, which facilitate more precise diabetes classification and newborn screening [7].

It is becoming increasingly evident that infection, gut microbiota, and nutritional factors play a significant role in the development of T1DM. The gut microbiota's potential role in T1DM pathogenesis has garnered interest due to its interactions with the immune system [2, 8]. Siljander et al.'s [9] review highlights key differences in the gut microbiome composition between T1DM patients and healthy individuals, including alterations in the Bacteroidetes/Firmicutes ratio, a reduction in butyrate-producing bacteria, and disruptions in short-chain fatty acid biosynthesis, contributing to a proinflammatory milieu. Animal studies have illustrated the interaction between the innate and adaptive immune systems and gut microbiota prior to T1DM onset [10]. However, the causality between gut microbiota dysbiosis and T1DM is yet to be fully elucidated.

HLA's role in gut microbiota structure, attributed to its antigen presentation function, is suggested by studies like those by Shahi et al. [11], which demonstrate HLA class II's direct impact on gut microbiota composition and its necessity for maintaining microbiome diversity. The influence of HLA genotype on IgA antibody selection against the microbiota, leading to distinct microbial communities that affect host susceptibility to enteric infections, has been indicated [12]. Furthermore, previous studies have also linked HLA heterozygosity with enhanced gut microbial diversity and function [13]. Subsequent studies have sought to establish associations between particular HLA genotypes and gut microbiota, particularly in autoimmune diseases such as ankylosing spondylitis, rheumatoid arthritis, and celiac disease [14, 15]. This is in light of the demonstrated impact of the absence and heterozygosity of HLA on gut microbiota. Significant differences in the structure of the gut microbiota were identified not only between spondylarthritis patients and healthy individuals, but also between HLA-B27-positive and -negative siblings of spondylarthritis patients [16, 17]. Furthermore, HLA-B27 transgenic rats exhibited a distinctive gut microbiota profile prior to the onset of arthritis, although divergent gut microbial alterations were observed across different genetic backgrounds [16, 18].

The current research on the association between HLA genotypes and gut microbiota in T1DM patients is limited [19, 20], with the study conducted focusing exclusively on adult T1DM patients with prolonged disease duration [19]. As highlighted in recent literature, notable variations in gut microbiota profiles were observed between pediatric and adult T1DM populations [21–23], and acknowledging the largely uncharted territory regarding the intricate relationship between specific HLA loci and gut microbiota composition, our study utilized whole-metagenome shotgun sequencing to investigate the gut microbiota profiles among T1DM patients with varying degrees of HLA-risk. Additionally, we endeavored to ascertain whether children possessing particular HLA loci linked to clinical presentations exhibit unique gut microbiota configurations.

## 2. Materials and Methods

### 2.1. Study Population

In this cross-sectional study, we recruited newly diagnosed T1DM patients, aged under 18 years old, with a disease duration of less than 1 month from nine geographically diverse cities in China, including Shanghai, Fuzhou, Nanchang, Suzhou, Jinan, Taiyuan, Changchun, Harbin, and Zhengzhou. The recruitment took place from January 2018 to December 2023. The diagnosis of T1DM was based on the criteria set forth by the International Society for Pediatric and Adolescent Diabetes (ISPAD) [24]. Cases under the age of 2 years, with other types of diabetes, other inherited metabolic disorders, serious chronic disorders, or incomplete crucial data were excluded. A total of 66 children with T1DM provided fecal samples for analysis. These participants were queried about their recent medication history and the history of infectious diseases and digestive tract disease. Fecal samples from individuals who received antibiotic or probiotic treatment within 1 month, or those with infectious diseases, chronic or acute gastrointestinal diseases, were not included in this study (Supporting Information 1: Figure S1). The healthy control group was matched with the subgroup of T1DM patients who donated fecal samples based on region, sex, age, and time of visit. This study was approved by the Ethics Committee of Children's Hospital of Fudan University (2016)210 and (2019)210. Prior to the collection of information and samples, written informed consent was obtained from the guardians of the children.

### 2.2. Sample Collection

Clinical data were meticulously collected for all participants, including sex, birth date, time of visit, HbA1c, and serum fasting C-peptide, through standardized questionnaires. For both the T1DM patients and the control group children, blood samples were obtained. Simultaneously, fecal samples were collected using dedicated sterile containers. Both blood and fecal samples were frozen and transported on dry ice to the Children's Hospital of Fudan University, and stored at −80°C until analysis.

### 2.3. Gut Microbiota Analysis

Details of DNA isolation, sequencing and analyzing procedures were described in the previous study [21]. In Brief, shotgun sequencing was conducted on the Illumina NovaSeq (Illumina Inc., San Diego, CA, USA) at Honsunbio Technology Co., Ltd. (Shanghai, China), utilizing NovaSeq Reagent Kits. Low-quality reads which length <50 bp or with a quality value <20 or had N bases, and reads aligned to the human genome were removed. Open reading frames (ORFs) from the assembled contigs were predicted by MetaGene (http://metagene.cb.k.u-tokyo.ac.jp/) [25].

### 2.4. HLA Sequencing and HLA-Risk Score Calculation

DNA was extracted from blood samples for the purpose of HLA sequencing. The HLA genes were sequenced by polymerase chain reaction sequence-based typing and analyzed according to the IMGT/HLA Database [26]. Following the identification of HLA alleles, a risk score was calculated for each individual based on the weight of different HLA loci, as previously described in the literature [7].

### 2.5. Statistics

The data were processed using R version 4.1.0 and GraphPad Prism version 9. The clinical data were described as numbers (proportions) for categorical variables and means (standard deviations) for continuous variables. The statistical significance of differences between two groups for normally distributed continuous variables was evaluated using Student's *t*-test. For variables that were not normally distributed, the Mann–Whitney *U* test was employed. Categorical variables were subjected to a comparison using the *χ*^2^ test. Spearman correlation analysis was employed to assess the relationship between clinical characteristics and HLA genotypes. A *p*-value of less than 0.05 was considered to be statistically significant.

A receiver operating characteristic analysis was conducted on the HLA-risk score, resulting in the calculation of Youden's index (AUC = 0.678, *p* < 0.001) and the determination of a cutoff value of 0.01. Consequently, children with an HLA score exceeding 0.01 were designated as belonging to the high-risk group, while those with a score below this threshold were classified as belonging to the low-risk group.

The indices of α-diversity and β-diversity, the taxonomic abundance, the linear discriminant analysis effect size analysis (LEfSe), and the Venn analysis were conducted using the R microeco package [27].

This study was reported in accordance with the STROBE guidelines (Supporting Information 2: Table S2).

## 3. Results

### 3.1. General Clinical Characteristics

A total of 106 children with newly diagnosed T1DM and 69 healthy control children were included in this study. Of the T1DM group, 57 (53.8%) were boys, while 42 (60.9%) of the control group were boys (*p*=0.355). The mean age of the T1DM group was 7.97 ± 3.30 years, closely matching that of the control group at 7.92 ± 2.90 years (*p*=0.920). The T1DM group exhibited significantly elevated HbA1c% levels in comparison to the control group (12.00% ± 2.06 % vs. 5.06% ± 0.51 %, *p* < 0.001). Additionally, the mean fasting C-peptide level of the T1DM group was 0.22 ± 0.17 μg/mL.

### 3.2. HLA Genotype Distribution

Referring to the Chinese HLA Common and Well-Documented catalog 2.2 (CCWD 2.2), the frequencies of common alleles for HLA-A, -B, -C, -DRB1, and -DQB1 in the control group were found to be highly comparable with those in the CCWD 2.2 database (Supporting Information 1: Table S1). This similarity indicates that the study population is representative of the broader genetic landscape documented in the CWD catalog. Furthermore, the frequency of well-known T1DM high-risk alleles, for example DRB1*⁣*^*∗*^0301 (*p* < 0.001), DRB1*⁣*^*∗*^0405 (*p* < 0.001), and DQB1*⁣*^*∗*^0201 (*p* < 0.001), was significantly higher in the T1DM comparing with the CCWD 2.2 database; while the frequence of the protective alleles, for example, DRB1*⁣*^*∗*^1501 (*p* < 0.001), was significantly lower in the T1DM group.

### 3.3. HLA Genotype Specific Clinical Characteristics

The average HLA-risk score for the T1DM group was significantly higher than that of the control group, with means of 0.41 ± 1.56 and −0.70 ± 1.73, respectively (*p* < 0.001). Utilizing a cutoff value of 0.01 for the HLA-risk score, 63 (59.4%) T1DM children were classified as high-risk compared to 21 (30.4%) healthy control children (*p* < 0.001), indicating a strong association between HLA-risk score and T1DM status. Among T1DM patients, there was no significant difference of age (7.64 ± 3.28 vs. 8.46 ± 3.30, *p*=0.211) and HbA1c (11.93% ± 1.87 % vs. 12.10% ± 2.33 %, *p*=0.693) between T1DM children with high and low HLA-risk (Table 1). However, children with high HLA-risk exhibited significantly lower fasting C-peptide levels compared to those with low HLA-risk (0.19 ± 0.14 μg/mL vs. 0.26 ± 0.19 μg/mL, *p*=0.029).

**Figure 1:**
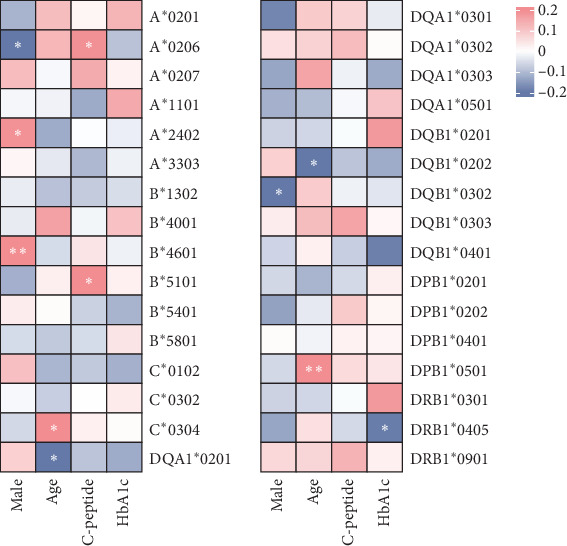
The association between HLA loci and clinical characteristics among newly onset type 1 diabetes children. *⁣*^*∗*^*p* < 0.05.

To delve deeper into the relationship between HLA genotypes and clinical manifestations at T1DM onset, a correlation analysis was conducted between HLA loci with a frequency higher than 10% and clinical characteristics (Figure 1). The analysis revealed that specific HLA loci were associated with clinical characteristics at onset. Notably, the onset age of T1DM was found to be delayed in children positive for HLA-C^*∗*^0304 and DPB1*⁣*^*∗*^0501 (Supporting Information 1: Figure S2), while HLA-DQA1*⁣*^*∗*^0201 and DQB1*⁣*^*∗*^0202 were negatively related with the onset age. After adjusting for the HLA-risk score and gender, we found that the C^*∗*^0304 locus, which is not included in the HLA-risk scoring system, still shows a positive correlation with the age of onset (*r* = 0.225, *p*=0.017). Furthermore, children with HLA-A^*⁣*^*∗*^^0206 and B^*⁣*^*∗*^^5101 tended to have higher fasting C-peptide levels at onset, while HLA DRB1*⁣*^*∗*^0405 was associated with lower HbA1c levels.

### 3.4. The Gut Microbiota Profile of Children With High- or Low-HLA-Risk Score

**Figure 2:**
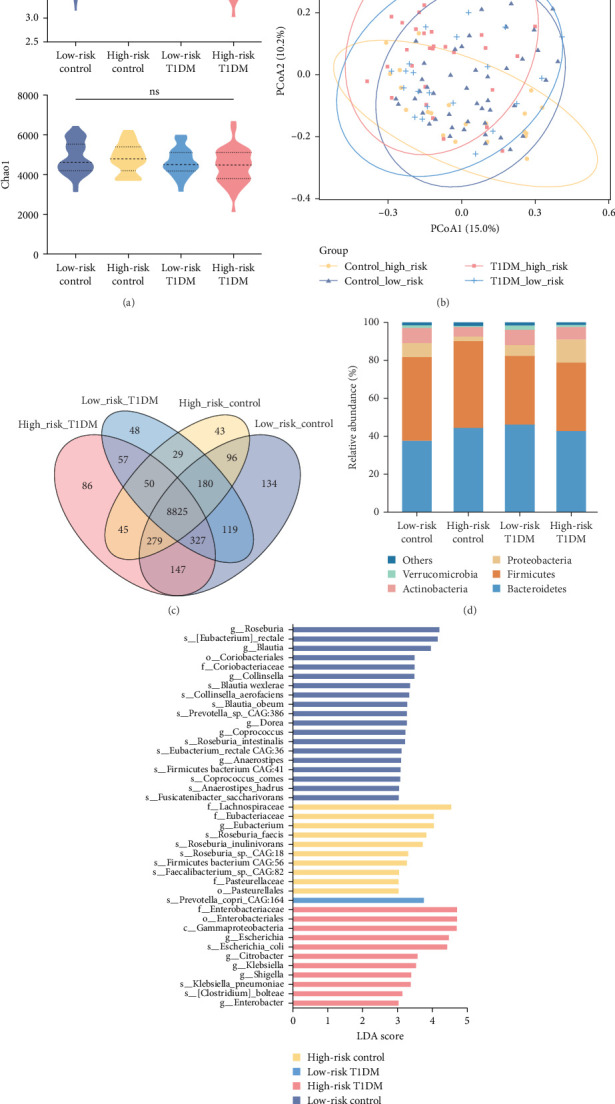
The characteristics of gut microbiota of type 1 diabetes children with high HLA-risk. (A) Shannon index and Chao1 index. (B) PCoA of gut microbiota among type 1 diabetes and control children with high- or low-risk. (C) OTU number in each group. (D) Composition of the gut microbiota at phylum level. (E) LEfSe comparison among type 1 diabetes and control children with high- or low-risk.

A metagenomic sequencing analysis was conducted on fecal samples from 57 children with T1DM and 69 healthy control children. The Shannon and Chao1 indices, which indicated the richness and diversity of the microbiota, were found to be similar between the T1DM and control groups, regardless of their risk level (Figure 2A), suggesting that the overall diversity and species richness of the gut microbiota are not directly affected by the HLA-risk status. Principal coordinates analysis (PCoA) demonstrated a significant divergence in microbial community structure among the four groups (PerMANOVA: *R*^2^ = 0.04696, *p*=0.001, Figure 2B). The low-risk control group and the high-risk control group exhibited significant differences in gut microbiota composition compared to the high-risk T1DM group (*R*^2^ = 0.0343, *p*=0.003; *R*^2^ = 0.0562, *p*=0.003). However, no significant differences were observed between the high-risk and low-risk control groups (*p*=0.113), or within the T1DM group between high-risk and low-risk categories (*p*=0.169). Additionally, no significant differences were found between the low-risk T1DM group and either the low-risk control group (*p*=0.113) or the high-risk control group (*p*=0.113).

Venn diagram analysis identified distinct operational taxonomic units (OUTs) unique to each HLA-risk group within the T1DM and control populations. Specifically, the high-risk T1DM group displayed 86 unique OUTs, contrasting with 48 unique OUTs found in the low-risk T1DM group (Figure 2C). Among the control group, the high-risk subset exhibited 43 unique OUTs, whereas the low-risk subset exhibited a higher number of unique OUTs, totaling 134. Although a trend was observed with T1DM cases showing a higher abundance of Firmicutes and a lower abundance of Bacteroidetes compared to control cases, these differences did not reach statistical significance (Figure 2D). The LEfSe analysis revealed an increase in the relative abundance of taxa from Lachnospiraceae, including *Roseburia*, *Blautia*, and *Dorea* in the low-risk control group. Conversely, an increase in the relative abundance of Enterobacteriaceae was observed in the high-risk T1DM group (Figure 2E).

### 3.5. Distinct Gut Microbiota Profile of HLA-C^*∗*^0304 Positive Children

Among loci positively associated with the onset age of T1DM, DPB1*⁣*^*∗*^0501 was previously identified as a protective locus of T1DM. However, no common characteristics of gut microbiota were observed among DPB1*⁣*^*∗*^0501-positive T1DM children and healthy control children. Consequently, our attention was drawn to HLA-C^*∗*^0304, which has not been extensively studied in relation to T1DM. In our study population, the positive rate of HLA-C^*∗*^0304 was significantly lower in the T1DM group than in the control group (13 [12.3%] vs. 18 [26.1%], *p*=0.019).

**Figure 3:**
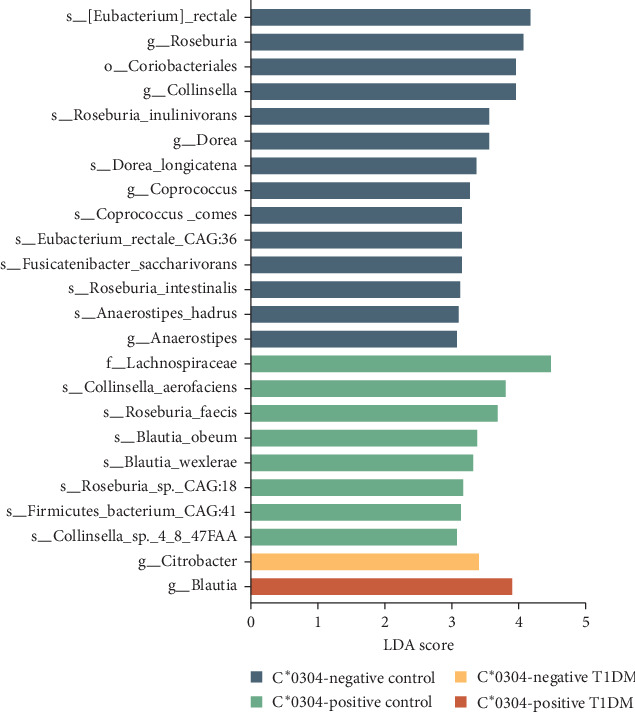
LEfSe comparison among type 1 diabetes and control children with or without HLA-C^*∗*^0304.

**Figure 4:**
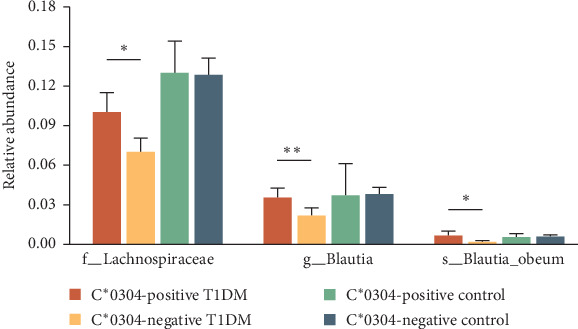
Differences in relative abundance of characteristic taxa among type 1 diabetes and healthy children with and without HLA-C^*∗*^0304. *⁣*^*∗*^*p* < 0.05; *⁣*^*∗∗*^*p* < 0.01.

The impact of a single HLA locus on the structure of the gut microbiota was found to be minimal. No significant differences were observed in α diversity or β diversity of the gut microbiota between HLA-C^*∗*^0304-positive and negative T1DM children. However, the abundance of *Blautia* was found to be higher in HLA-C^*∗*^0304-positive individuals (Figure 3). Moreover, patients with T1DM who were HLA-C^*∗*^0304 negative exhibited a relatively higher abundance of opportunistic pathogens, namely *Citrobacter*, which belongs to the Enterobacteriaceae family. A significantly higher relative abundance of Lachnospiraceae (*p*=0.039), *Blautia* (*p*=0.005), and *Blautia obeum* (*p*=0.023) was observed in HLA-C^*∗*^0304-positive T1DM children compared to HLA-C^*∗*^0304-negative T1DM children (Figure 4).

## 4. Discussion

Although adult-onset T1DM seems to have weaker familial aggregation and lower heritability than childhood-onset T1DM [28], the complex interplay among specific HLA loci, the gut microbiota's composition, and the clinical presentation of the disease necessitates further investigation in the context of pediatric T1DM. Our multicenter cross-sectional study elucidates the hypothesis that distinct HLA genotypes may exert a significant influence on the trajectory of T1DM by modulating the gut microbiota. Our findings suggest a potential role for the HLA-C^*∗*^0304 allele in the etiology of T1DM among children. Notably, these alleles exhibited a robust association with the abundance of butyrate-producing bacteria, which may be crucial in the pathogenesis of T1DM.

The HLA genes, which represent the primary genetic risk factors, offer a promising avenue for the prognostication and diagnostic refinement of T1DM [7, 29, 30]. The age at which T1DM is diagnosed is influenced by the pace of progression during its preclinical phases [1], children with high-risk HLA genotypes tend to develop T1DM at a younger age than those with neutral or protective HLA genotypes [31].

The timing of T1DM onset appears to be influenced by specific HLA genotypes, with growing evidence pointing to a role for both class I and class II molecules [32, 33]. Certain protective HLA class II haplotypes, including DRB1*⁣*^*∗*^1501-DQB1*⁣*^*∗*^0602 and DRB1*⁣*^*∗*^0701-DQB1*⁣*^*∗*^0303, have been found to exhibit a lower prevalence in children who experience an earlier onset of T1DM [34]. While the clinical implications of HLA class I variations have received less attention compared to those of class II, studies among Chinese T1DM patients have found that HLA-A^*⁣*^*∗*^^11:01:01 is positively associated with onset age, while HLA-C^*∗*^15:02:01 is negatively associated with onset age [6]. Our study's results demonstrated a positive association between the C^*∗*^0304 and DPB1*⁣*^*∗*^0501 and the age of onset. In studies conducted among the Han population, HLA-DPB1*⁣*^*∗*^0501 was identified as a protective locus, with no adjustment made for potential confounding factors [35, 36]. However, the available evidence on the association between T1DM and HLA-C^*∗*^0304 is limited. The Type 1 Diabetes Genetics Consortium reported that HLA-C^*∗*^0304 was protective [5], meanwhile, another study among the Chinese population found that HLA-C^*∗*^0304 was considered as protective loci [37], suggesting a potential genetic predisposition within this demographic that could influence the development of T1DM.

While HLA class II molecules present exogenous peptides to CD4^+^ T cells and are central to the initiation of islet autoimmunity, class I molecules present endogenous peptides to CD8^+^ cytotoxic T cells and are more directly involved in shaping the inflammatory environment that drives β-cell destruction [2]. The mechanisms through which HLA-C^*∗*^0304 delays T1DM onset remain unclear, but its influence on the CD8^+^ T cell response suggests a potential role in modulating the tempo of islet autoimmunity. Beyond direct immune interactions, HLA genotypes may influence T1DM onset indirectly by shaping the gut microbiota and mucosal immune responses. For example, in individuals with the HLA-DR3/DR4 haplotype, the presence of serum antibodies against specific intestinal bacteria correlates with an increased risk of subsequent T1DM diagnosis [38]. This DR3/DR4-linked association of anti-commensal antibodies suggests a link between HLA genotype, gut microbiota, and islet autoimmunity. The All Babies in Southeast Sweden cohort study has indicated that individuals with protective HLA genotypes for T1DM display a higher prevalence of *Intestinibacter* and *Romboutsia* species in their gut microbiota by the age of one, suggesting a potential microbiota-mediated mechanism for immune protection [20]. Our study revealed that the novel protective effect of HLA-C^*∗*^0304 may be achieved by affecting the abundance of specific taxa, particularly *Blautia*, in the gut microbiota. *Blautia*, as a member of the Lachnospiraceae family, is recognized for its ability to impede the intestinal colonization of pathogenic bacteria, synthesize acetic acid, and enhance glucose and lipid metabolism [39]. This bacterial genus also mitigates intestinal inflammation by activating regulatory T cells and generating short-chain fatty acids. Its diminished presence in inflammatory bowel disease underscores its role in immune modulation and inflammation [40]. Its enrichment in HLA-C^*∗*^0304-positive individuals with T1DM may contribute to delayed disease onset by enhancing gut barrier integrity and immune regulation. These findings suggest that HLA-driven modulation of the gut microbiome may be a critical intermediary linking genetic risk with immune activity, providing a logical bridge to understanding the broader role of HLA–microbiota interactions in autoimmune pathogenesis.

The interplay between host genetics and gut microbiota is increasingly recognized as a contributing factor in the development of T1DM in children. Grieneisen et al. [41] demonstrated the near-universal heritability of gut microbiome traits in baboons, suggesting that host genetics exert a pervasive, albeit modest, influence on microbial composition, though the degree of heritability varies with environmental factors. However, the limited replicability of findings in human studies underscores the challenges in pinpointing specific host genetic loci, with only LCT and ABO consistently linked to microbiome variation [42]. Building on this, Berryman et al. [43] found that infants with high-T1DM-risk HLA genotypes (DR3–DQ2.5 and DR4–DQ8) had gut microbiomes enriched in proinflammatory functions, while protective genotypes like DR15–DQ6.2 were linked to more stable, metabolic profiles. DR3-associated microbiota also showed reduced binding affinity to flagellin, potentially allowing sustained innate immune activation. These findings align with our findings and suggest that HLA genotypes shape not only the taxonomic structure but also the immunological function of the gut microbiota, contributing to early-life dysbiosis and the autoimmune cascade.

Additional evidence from other autoimmune conditions further supports the HLA–microbiota interaction model. For example, the HLA-B27 allele, strongly linked to ankylosing spondylitis, has been shown to alter gut microbiota and metabolite profiles in both humans and animal models, contributing to cytokine dysregulation [44–46]. Likewise, HLA-A29, which is exclusively associated with birdshot retinochoroidopathy, has been linked to distinct microbiota profiles, including a higher prevalence of *Clostridium difficile* [47]. In contrast, the 8.1 ancestral haplotype (AH8.1), despite its strong association with multiple autoimmune diseases such as T1DM and celiac disease, appears to have minimal impact on gut microbiota composition [48]. The mechanisms by which HLA genotypes influence microbiota are not yet fully understood, current hypotheses suggest that differential epitope presentation, mucosal immunity modulation and metabolic niche construction collectively shape T cell and antibody responses that select for specific gut microbial communities. The protective role of HLA-C^*∗*^0304 in T1DM is suggested by a diminished frequency of deleterious bacterial strains and an upregulation of beneficial microbiota. Prospective studies on the modulation of gut microbiota by HLA alleles and other genetic factors could pave the way for novel interventions in autoimmune conditions, including T1DM.

It is important to acknowledge the limitations inherent in this study. First, the strong linkage disequilibrium within the HLA region precluded the determination of additional HLA alleles associated with HLA-C^*∗*^0304. However, extensive studies encompassing Western and Chinese populations have not revealed a significant linkage disequilibrium between established high-risk HLA loci for T1DM and HLA-C^*∗*^0304 [5, 6]. Thus, we surmise that our results are not confounded by other T1DM-associated HLA loci. Second, the sample size of the current study is modest. However, its representativeness and statistical sufficiency are supported by the geographical representation covering eight different regions all around China, the comparability of high-frequency HLA loci with Chinese population and the plateaued species accumulation curve of gut microbiota (Supporting Information 1: Figure S3), confirming the sample size is sufficient for the reported gut microbiota comparisons. Third, the cross-sectional design of our study precluded causal inference and hampered our capacity to track the dynamic changes in gut microbiota structure among children with diverse HLA genotypes as T1DM progresses. Longitudinal studies—especially prospective birth cohorts of high-risk infants—are warranted to delineate the temporal interplay between HLA genotype, microbiome maturation, and islet autoimmunity, thereby clarifying whether HLA-C^*∗*^0304 actively shapes a protective microbiome or merely marks an epiphenomenon. Such cohorts will also allow parallel tracking of microbiota shifts and islet autoimmunity across diverse HLA backgrounds. Complementarily, gnotobiotic HLA-humanized mouse models and single-strain intervention experiments are needed to test causality under controlled conditions. Finally, the absence of metabolomic data, particularly fecal short-chain fatty acid quantification, constrained mechanistic interpretation. Beyond short-chain fatty acid, *Blautia* can modulate amino-acid-derived metabolites, carbohydrate metabolism, and influence the function immune cells and the expression of cytokines [49–53]. Future cohort studies should, therefore, integrate comprehensive targeted metabolomics and immune profiles, and gnotobiotic single-strain experiments to dissect these multifaceted, *Blautia*-specific mechanisms in the context of islet autoimmunity.

## 5. Conclusions

In conclusion, our study in pediatric T1DM suggests that the presence of HLA-C^*∗*^0304 may be associated with a delay in the onset of T1DM. This association could be due to the maintenance of a more abundant population of beneficial bacteria, such as *Blautia*, in the gut microbiota. Although the specific mechanisms behind this relationship are not yet fully clarified, our results hint at the possibility that genetic factors subtly shape the gut microbiota's composition. Moving forward, prospective birth-cohort studies that concurrently monitor microbiota maturation and islet-autoantibody emergence across diverse HLA backgrounds will be essential to confirm and extend these findings. This observation could potentially inform the development of future strategies aimed at preventing and managing T1DM.

## Figures and Tables

**Table 1:** Clinical characteristics of T1DM children with high or low HLA-risk.

Characteristics	Control (*n* = 69)	T1DM			*p*-Value*⁣*^*∗*^
		Total (*n* = 106)	High HLA-risk (*n* = 63)	Low HLA-risk (*n* = 43)	
Male (%)	42 (60.9%)	57 (53.8%)	29 (46.0%)	28 (65.1%)	0.053
Age, years	7.92 ± 2.90	7.97 ± 3.30	7.64 ± 3.28	8.46 ± 3.30	0.211
HbA1c (%)	5.06 ± 0.51	12.00 ± 2.06	11.93 ± 1.87	12.10 ± 2.33	0.693
C-peptide (μg/mL)	Not available	0.22 ± 0.17	0.19 ± 0.14	0.26 ± 0.19	0.029
HLA-risk score	−0.70 ± 1.73	0.41 ± 1.56	1.37 ± 1.22	−1.00 ± 0.67	<0.001

*⁣* ^*∗*^Comparison between the high-HLA-risk T1DM group and the low-HLA-risk T1DM group.

## Data Availability

The data that support the findings of this study are available from the corresponding author upon reasonable request.

## Supporting Information

Supporting Information 1 Figure S1. Flow chart. Figure S2. The different clinical characteristics of T1DM with and without specific HLA loci. Table S1. HLA genotype distribution.

Supporting Information 2 Table S2. STROBE-checklist.
