# Supplementary material for: HLA-C⁣∗0304 Associates With Beneficial Gut Microbiota and Later Onset of Type 1 Diabetes in Pediatric Cohorts
Source: Pediatr Diabetes. 2025 Oct 28;2025:3013063. doi: 10.1155/pedi/3013063 (PMC12585876; doi:10.1155/pedi/3013063)
Supplement: Supporting Information 1 — Figure S1. Flow chart. Figure S2. The different clinical characteristics of T1DM with and without specific HLA loci. Table S1. HLA genotype distribution. [file 3013063.f1.docx]

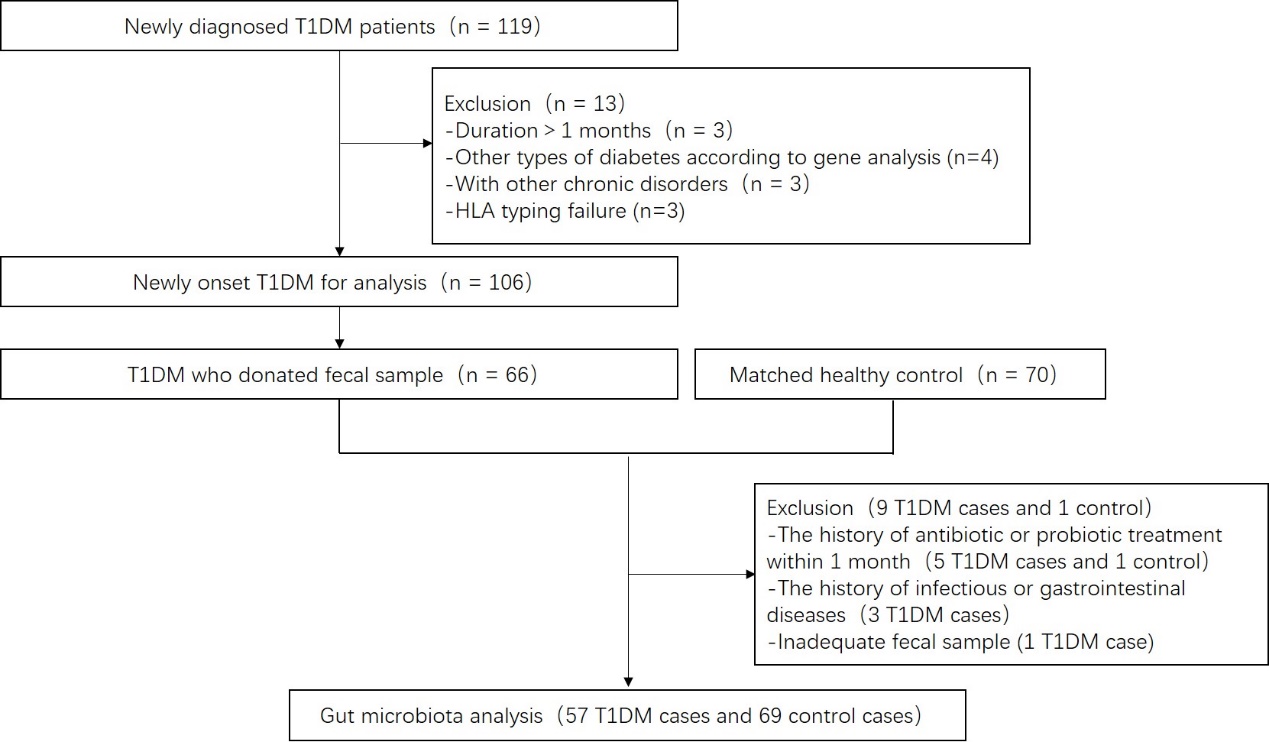


**Supplementary Fig S1.** Flow chart


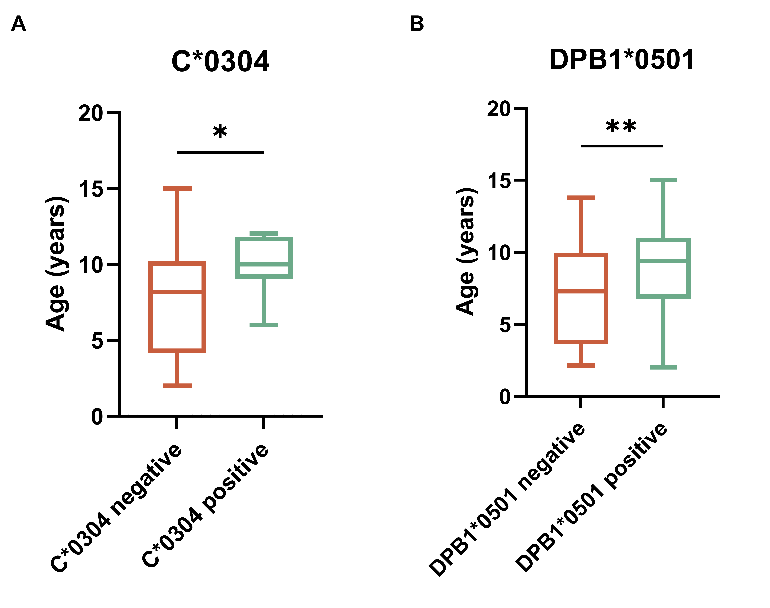


**Supplementary Fig S2.** The different clinical characteristics of T1DM with and without specific HLA loci

* *p* < 0.05, ** *p* < 0.01


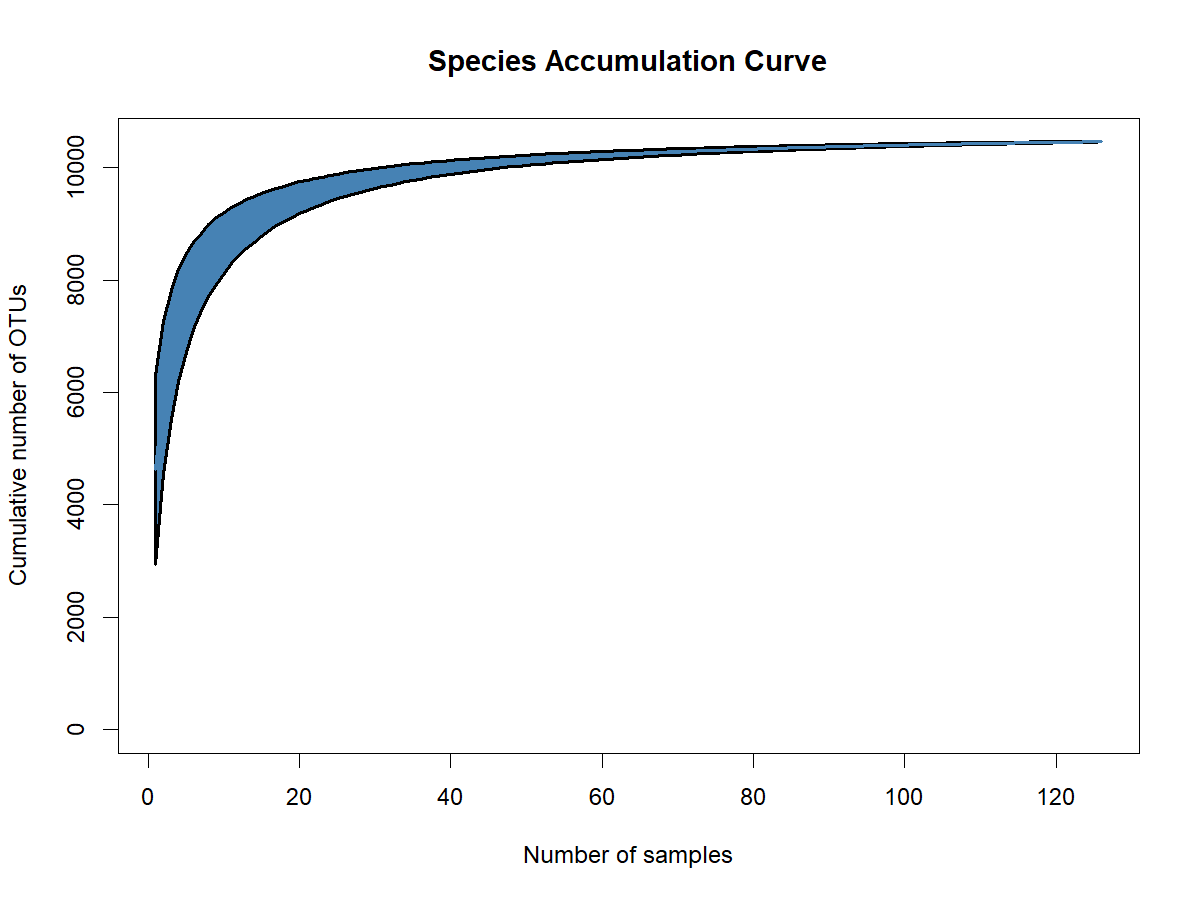


**Supplementary Fig S3.** **Species accumulation curve.**

The curve was computed by random sample-based rarefaction (1,000 permutations) across all 126 fecal samples.

**Supplementary Table S1** HLA Genotype Distribution

|  |  | This study  control group  (2n = 138) | This study  T1DM group  (2n = 212) | CCWD2.2 | P value  Control  *vs.* CCWD2.2 | P value  T1DM  *vs.* CCWD2.2 |
| --- | --- | --- | --- | --- | --- | --- |
| A* |  |  |  |  |  |  |
|  | **01:01** | 3 (2.17) | 3 (1.42) | 60182 (3.58) | 0.495# | 0.090 |
|  | **02:01** | 22 (15.94) | 35 (16.51) | 201889 (12.01) | 0.156 | **0.044** |
|  | **02:03** | 4 (2.90) | 6 (2.83) | 58460 (3.48) | 1.000# | 0.606 |
|  | **02:06** | 4 (2.90) | 18 (8.49) | 87533 (5.21) | 0.222 | **0.032** |
|  | **02:07** | 16 (11.59) | 19 (8.96) | 141843 (8.44) | 0.183 | 0.785 |
|  | **03:01** | 5 (3.62) | 4 (1.89) | 49769 (2.96) | 0.610# | 0.356 |
|  | **11:01** | 24 (17.39) | 22 (10.38) | 351779 (20.93) | 0.306 | **< 0.001** |
|  | **11:02** | 2 (1.45) | 3 (1.42) | 29440 (1.75) | 1.000# | 1.000# |
|  | **24:02** | 19 (13.77) | 42 (19.81) | 260906 (15.53) | 0.569 | 0.085 |
|  | **26:01** | 4 (2.90) | 5 (2.36) | 47750 (2.84) | 0.799# | 0.672 |
|  | **30:01** | 5 (3.62) | 10 (4.72) | 99311 (5.91) | 0.255 | 0.461 |
|  | **31:01** | 5 (3.62) | 2 (0.94) | 54969 (3.27) | 0.808# | 0.057 |
|  | **32:01** | 2 (1.45) | 4 (1.89) | 22372 (1.33) | 0.707# | 0.371# |
|  | **33:03** | 17 (12.32) | 30 (14.15) | 137528 (8.18) | 0.076 | **0.002** |
| B* |  |  |  |  |  |  |
|  | **07:02** | 1 (0.72) | 0 (0) | 35608 (2.12) | 0.378# | **0.026#** |
|  | **13:01** | 9 (6.52) | 3 (1.42) | 84569 (5.03) | 0.424 | **0.016** |
|  | **13:02** | 4 (2.9) | 12 (5.66) | 105324 (6.27) | 0.103 | 0.715 |
|  | **15:01** | 1 (0.72) | 7 (3.3) | 80227 (4.77) | **0.026** | 0.315 |
|  | **15:02** | 4 (2.9) | 2 (0.94) | 60295 (3.59) | 1.000# | **0.038** |
|  | **15:11** | 4 (2.9) | 4 (1.89) | 30905 (1.84) | 0.327# | 0.799# |
|  | **15:18** | 0 (0) | 5 (2.36) | 22896 (1.36) | 0.271# | 0.220# |
|  | **40:06** | 3 (2.17) | 11 (5.19) | 54072 (3.22) | 0.806# | 0.104 |
|  | **44:03** | 6 (4.35) | 2 (0.94) | 46152 (2.75) | 0.285 | 0.108 |
|  | **46:01** | 11 (7.97) | 24 (11.32) | 172700 (10.28) | 0.372 | 0.617 |
|  | **48:01** | 2 (1.45) | 5 (2.36) | 41412 (2.46) | 0.779# | 0.921 |
|  | **51:01** | 6 (4.35) | 11 (5.19) | 94565 (5.63) | 0.514 | 0.782 |
|  | **51:02** | 1 (0.72) | 0 (0) | 17729 (1.06) | 1.000# | 0.181# |
|  | **52:01** | 3 (2.17) | 4 (1.89) | 49968 (2.97) | 0.802# | 0.352 |
|  | **54:01** | 3 (2.17) | 21 (9.91) | 52432 (3.12) | 0.804# | **< 0.001** |
|  | **55:02** | 5 (3.62) | 4 (1.89) | 41714 (2.48) | 0.400# | 0.577 |
|  | **57:01** | 1 (0.72) | 1 (0.47) | 19598 (1.17) | 1.000# | 0.527# |
|  | **58:01** | 13 (9.42) | 38 (17.92) | 102978 (6.13) | 0.107 | **< 0.001** |
| C* |  |  |  |  |  |  |
|  | **01:02** | 19 (13.77) | 47 (22.17) | 179483 (15.89) | 0.496 | **0.012** |
|  | **03:02** | 13 (9.42) | 36 (16.98) | 67014 (5.93) | 0.083 | **< 0.001** |
|  | **03:03** | 6 (4.35) | 12 (5.66) | 78335 (6.93) | 0.232 | 0.465 |
|  | **03:04** | 21 (15.22) | 14 (6.6) | 112096 (9.92) | **0.037** | 0.106 |
|  | **04:01** | 10 (7.25) | 5 (2.36) | 65172 (5.77) | 0.457 | **0.033** |
|  | **04:03** | 3 (2.17) | 2 (0.94) | 11488 (1.02) | 0.176 | 1.000# |
|  | **06:02** | 6 (4.35) | 18 (8.49) | 100031 (8.85) | 0.062 | 0.852 |
|  | **07:02** | 25 (18.12) | 25 (11.79) | 171619 (15.19) | 0.339 | 0.168 |
|  | **08:01** | 6 (4.35) | 25 (11.79) | 96496 (8.54) | 0.078 | 0.090 |
|  | **12:02** | 6 (4.35) | 3 (1.42) | 35383 (3.13) | 0.333# | 0.151 |
|  | **12:03** | 1 (0.72) | 3 (1.42) | 21799 (1.93) | 0.529 | 0.803# |
|  | **14:02** | 3 (2.17) | 9 (4.25) | 48357 (4.28) | 0.222 | 0.980 |
|  | **14:03** | 4 (2.9) | 0 (0) | 11482 (1.02) | 0.053# | 0.286# |
|  | **15:02** | 4 (2.9) | 0 (0) | 37813 (3.35) | 1.000# | **0.007** |
| DRB1* |  |  |  |  |  |  |
|  | **01:01** | 2 (1.45) | 3 (1.42) | 34661 (2.06) | 1.000# | 0.806# |
|  | **03:01** | 6 (4.35) | 65 (30.66) | 85936 (5.11) | 0.683 | **< 0.001** |
|  | **04:03** | 2 (1.45) | 1 (0.47) | 26167 (1.56) | 1.000# | 0.272 |
|  | **04:05** | 8 (5.8) | 27 (12.74) | 80766 (4.81) | 0.586 | **< 0.001** |
|  | **04:06** | 3 (2.17) | 0 (0) | 43087 (2.56) | 1.000# | **0.018** |
|  | **07:01** | 9 (6.52) | 14 (6.6) | 161566 (9.61) | 0.218 | 0.137 |
|  | **08:03** | 9 (6.52) | 2 (0.94) | 105751 (6.29) | 0.912 | **0.001** |
|  | **09:01** | 16 (11.59) | 67 (31.6) | 247648 (14.74) | 0.298 | **< 0.001** |
|  | **10:01** | 1 (0.72) | 0 (0) | 26635 (1.58) | 0.730# | 0.087# |
|  | **11:01** | 11 (7.97) | 2 (0.94) | 94716 (5.64) | 0.234 | **0.003** |
|  | **12:01** | 5 (3.62) | 5 (2.36) | 40796 (2.43) | 0.394# | 0.984 |
|  | **12:02** | 18 (13.04) | 0 (0) | 146440 (8.71) | 0.071 | **< 0.001** |
|  | **13:01** | 3 (2.17) | 0 (0) | 24454 (1.46) | 0.458# | 0.081# |
|  | **13:02** | 9 (6.52) | 1 (0.47) | 55569 (3.31) | 0.050# | **0.021** |
|  | **14:05** | 4 (2.9) | 0 (0) | 36925 (2.2) | 0.551# | **0.017#** |
|  | **14:54** | 2 (1.45) | 0 (0) | 40705 (2.42) | 0.778# | **0.022** |
|  | **15:01** | 8 (5.8) | 3 (1.42) | 195013 (11.6) | **0.033** | **< 0.001** |
|  | **15:02** | 7 (5.07) | 5 (2.36) | 52822 (3.14) | 0.212# | 0.513 |
|  | **16:02** | 5 (3.62) | 2 (0.94) | 51852 (3.09) | 0.620# | 0.071 |
| DQB1* |  |  |  |  |  |  |
|  | **02:01** | 6 (4.35) | 65 (30.66) | 52717 (4.93) | 0.750 | **< 0.001** |
|  | **02:02** | 8 (5.8) | 14 (6.6) | 81232 (7.6) | 0.423 | 0.583 |
|  | **03:01** | 39 (28.26) | 6 (2.83) | 225256 (21.09) | **0.039** | **< 0.001** |
|  | **03:02** | 8 (5.8) | 23 (10.85) | 61374 (5.75) | 0.979 | **0.001** |
|  | **03:03** | 19 (13.77) | 65 (30.66) | 169687 (15.88) | 0.497 | **< 0.001** |
|  | **04:01** | 6 (4.35) | 23 (10.85) | 48083 (4.5) | 0.931 | **< 0.001** |
|  | **04:02** | 2 (1.45) | 0 (0) | 13426 (1.26) | 0.693# | 0.121# |
|  | **05:01** | 7 (5.07) | 5 (2.36) | 49542 (4.64) | 0.808 | 0.115 |
|  | **05:02** | 8 (5.8) | 6 (2.83) | 77912 (7.29) | 0.499 | **0.012** |
|  | **05:03** | 5 (3.62) | 1 (0.47) | 44741 (4.19) | 0.740 | **0.007** |
|  | **06:01** | 15 (10.87) | 2 (0.94) | 109306 (10.23) | 0.805 | **< 0.001** |
|  | **06:02** | 4 (2.9) | 1 (0.47) | 81403 (7.62) | **0.037** | **< 0.001** |
|  | **06:03** | 3 (2.17) | 0 (0) | 15747 (1.47) | 0.461# | 0.082# |
|  | **06:04** | 5 (3.62) | 1 (0.47) | 15340 (1.44) | 0.050# | 0.381# |
|  | **06:09** | 3 (2.17) | 0 (0) | 18080 (1.69) | 0.509# | 0.056# |

# Using Fisher's precision probability test.
